# Implementation of Arithmetic Functions on a Simple and Universal Molecular Beacon Platform

**DOI:** 10.1002/advs.201500054

**Published:** 2015-04-14

**Authors:** Hailong Li, Shaojun Guo, Qinghui Liu, Lidong Qin, Shaojun Dong, Yaqing Liu, Erkang Wang

**Affiliations:** ^1^ State Key Laboratory of Electroanalytical ChemistryChangchun Institute of Applied ChemistryChangchun130022China; ^2^ Graduate School of the Chinese Academy of SciencesBeijing100039China; ^3^ Department of NanomedicineHouston Methodist Research InstituteHoustonTX77030USA; ^4^ Department of Cell and Developmental BiologyWeill Medical College of Cornell UniversityNY10065USA

**Keywords:** adders, biomaterials, DNA nanotechnology, molecular computing, subtractors

## Abstract

**Diverse advanced logic circuits** are fabricated to implement arithmetic functions based on a simple and single molecular beacon platform, including half adder, half subtractor, full adder, full subtractor, and a digital comparator. Dual fluorescence outputs are generated in parallel and a constant threshold value is set to build all the logic circuits. The developed enzyme‐free DNA system provides a novel prototype for the design of high‐level molecular logic circuits on a biomolecular platform.

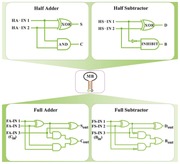

Looking through the history of humanity with respect to count and computing, electronic computers appeared just about 60 years ago, exhibiting unprecedented performance and thus revolutionizing computation. However, electronic computers mark neither the beginning nor the end of the history of computation due to its limits. Molecular computing is a fascinating area of research that involves molecular‐level data processing and has projected chemistry onto the forefront of information and technology.^1^, ^2^, ^3^, ^4^, ^5^, ^6^, ^7^, ^8^, ^9^, ^10^, ^11^, ^12^ In recent years, molecular logic gates exhibit great potential to perform computation on nanometer scale and have drawn increasing attention.^1^ Due to its novelty and inherent merits, various logic functions have been simulated on the molecular level since the first molecular AND logic gate.^8^, ^11^, ^13^ Not only sophisticated synthetic molecules but also biomolecules, such as proteins, enzymes, DNA, and RNA, have been exploited for information processing.^11^, ^14^, ^15^ DNA is considered an outstanding engineering material for addressing the central problems of molecular computing by virtue of its inherent merits, including easy synthesis, structural simplicity, high flexibility, sufficient sequence design space, and predictable molecular behavior.^16^, ^17^ To date, great efforts have been made to construct various fundamental DNA‐based logic gates.^18^, ^19^, ^20^ However, advanced logic circuits are rarely achieved at the molecular level due to the complexity of the process, which requires the integration of at least two kinds of logic gates with multiple inputs and outputs.^15^, ^21^ Although complex high‐level logic circuits are critical to processors, construction of these circuits at the molecular level is still a great challenge.^22^, ^23^

To make further advancements in molecular computation, we constructed a series of advanced logic circuits in proof‐of‐principle experiments using DNA to execute multiple arithmetic functions, including half adder, half subtractor, full adder, full subtractor, and digital comparator.^24^ These DNA networks are endowed with excellent biocompatibility compared with those based on complex synthetic organic molecules.^25^, ^26^, ^27^, ^28^, ^29^, ^30^, ^31^, ^32^, ^33^, ^34^, ^35^, ^36^ Another advantage over some previous networks lies in its multiple signal generation which occurs in parallel while sharing the same DNA‐based platform, not by a simple combination of independent logic gates to form desired circuits based on different platforms.^31^, ^37^, ^38^, ^39^, ^40^ Furthermore, from a potential applications point of view, it is important to construct multicomponent devices on a single biomolecular platform to meet the requirements of increased computational complexity.^41^, ^42^, ^43^, ^44^ Here, for the first time, all complex logic circuits to perform arithmetic functions share the same DNA‐based platform and a constant threshold setpoint, eliminating the need to use enzymes or DNAzymes to increase specificity.^45^, ^46^, ^47^ The versatility and power of molecular computing based on DNA here indicate its promising applications in solving complex combinatorial problems on the molecular level. We expect that our present DNA‐based molecular computing platform will find great potential in disease diagnosis and therapy due to its biocompatibility.^48^, ^49^, ^50^, ^51^

A half adder is an essential component of a digital signal processor to add two binary digits (bits) and can be implemented by integrating an AND and an XOR (XOR: exclusive OR) logic gate in parallel.^52^ In our investigation, the two required logic gates were constructed on a simple and universal DNA molecular beacon (MB) platform, terminally labeled with 6‐carboxyfluorescein (FAM) and Dabcyl and triggered by the same set of inputs. **Figure** **1**A illustrates the principle of the present half adder operation based on structural change induced by DNA hybridization (note that all DNA sequences described in the manuscript are available in the Supporting Information). The logic circuit of a half adder is shown in Figure 1B. The MB produces weak or strong FAM fluorescence depending on whether it is in a closed or open state,^53^ serving as one signal output. *N*‐Methylmesoporphyrin IX (NMM) is used as a second fluorescent reporter, generating enhanced fluorescence upon formation of a G‐4 (G‐quadruplex)/NMM complex.^54^, ^55^, ^56^ Distinct FAM and NMM fluorescence emission spectra can be observed in response to various combinations of inputs HA‐IN 1 and HA‐IN 2. As shown in Figure 1A, HA‐IN 1 and HA‐IN 2 are both able to open the MB, leading to the separation of FAM from the Dabcyl quencher and, thus, strong FAM fluorescence (curves b and c in Figure 1C). A single‐base mismatch is embedded in each MB‐HA‐IN duplex to decrease the hybridization energy to implement further interactions. The mismatched base is indicated with lower case letter in the input sequences. In addition, in the presence of MB and either input alone, there is no apparent effect on NMM fluorescence due to the lack of G‐quadruplex formation (curves b and c in Figure 1D). In the presence of both inputs together, hybridization between HA‐IN 1 and HA‐IN 2 takes priority over their respective interactions with MB. In this case, weak FAM fluorescence can be observed (curve d in Figure 1C). The hybridization between the two inputs facilitates G‐quadruplex formation from G‐rich splits GGGT at the 5′ end of HA‐IN 1 and TGGGTGGGTGGG at the 3′ end of HA‐IN 2 (Supporting Information, Half adder: Design of DNA sequences).^57^ Due to the formation of G‐4/NMM, strongly enhanced NMM fluorescence appears (curve d in Figure 1D). The interactions among the designed DNA strands were validated by native polyacrylamide gel electrophoresis (PAGE, see Figure S1, Supporting Information). The normalized fluorescence intensities of FAM and NMM are shown in Figure 1E. The presence and absence of each input are defined as input “1” and “0,” respectively. The output is defined as “1” or “0” when the fluorescence signal is above or below the threshold value 0.4, respectively. The definitions are available for all the following logic operations (half subtractor, full adder, full subtractor, and digital comparator). Thus, a half adder is achieved on proof‐of‐principle according to the obtained truth table (Figure 1F). Triggered by the same set of inputs (HA‐IN 1 and HA‐IN 2) to the MB platform, NMM‐related AND and FAM‐related XOR gates were produced in parallel, coding for the CARRY (C) and SUM (S) digits, respectively (Figure 1B,F). The digits shown in Figure 1F stand for the binary realization of 0 + 0 = 0, 1 + 0 = 1, and 0 + 1 = 1. In the case of 1 + 1, the SUM is 0, but the CARRY is set to 1, yielding 1 + 1 = 2.^26^

**Figure 1:**
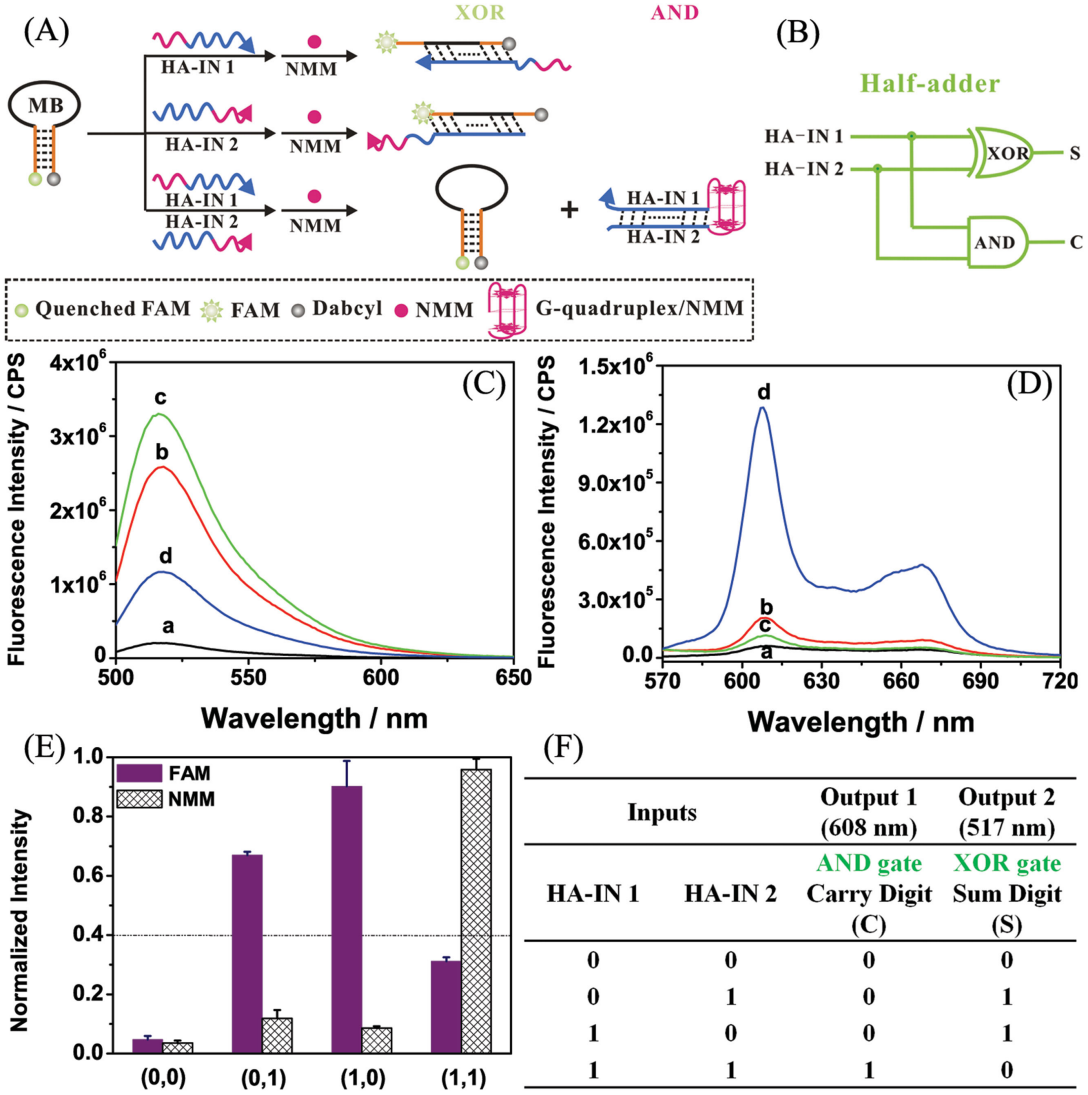
A) Schematic illustration of molecular‐scale implementation of a half adder based on DNA hybridization. B) Diagram of electronic half‐adder logic circuit. C,D) Fluorescence emission spectra of FAM (C) and NMM (D) in the presence of a) MB; b) MB + HA‐IN 2; c) MB + HA‐IN 1; d) MB + HA‐IN 1 + HA‐IN 2. E) Normalized fluorescence intensities of FAM and NMM outputs. F) Truth table for the half adder operation compiled from NMM‐based AND and FAM‐based XOR logic gates. FAM, 6‐carboxyfluorescein; NMM, *N*‐methyl mesoporphyrin IX; MB, molecular beacon; HA‐IN, half‐adder input.

A half‐subtractor operation was also pursued based on the same MB platform and two new designed inputs (HS‐IN 1 and HS‐IN 2). The principle is illustrated in **Figure** **2**A. A half subtractor is composed of parallel XOR and INHIBIT (INH) logic gates to produce DIFFERENCE (D) and BORROW (B) digits, respectively (Figure 2B).^52^ Here, FAM fluorescence (Figure 2C) was employed as output signal to construct the XOR gate, whereas NMM output (Figure 2D) was used to build the INH gate. The sequence design, discussion of changes in fluorescence with different input combinations, and PAGE analysis (Figure S2) are available in the Supporting Information. After normalization of FAM and NMM fluorescence intensities (Figure 2E), the obtained truth table (Figure 2F) clearly indicates parallel construction of FAM‐based XOR and NMM‐based INH logic gates, producing the D and B digits, respectively. Subtractions in a half subtractor are performed as follows (HS‐IN 1 – HS‐IN 2): 0 – 0 = 0, 1 – 0 = 1. 0 – 1 results in high borrow so the equation becomes 2 – 1 = 1, and finally, 1 – 1 = 0.^26^

**Figure 2:**
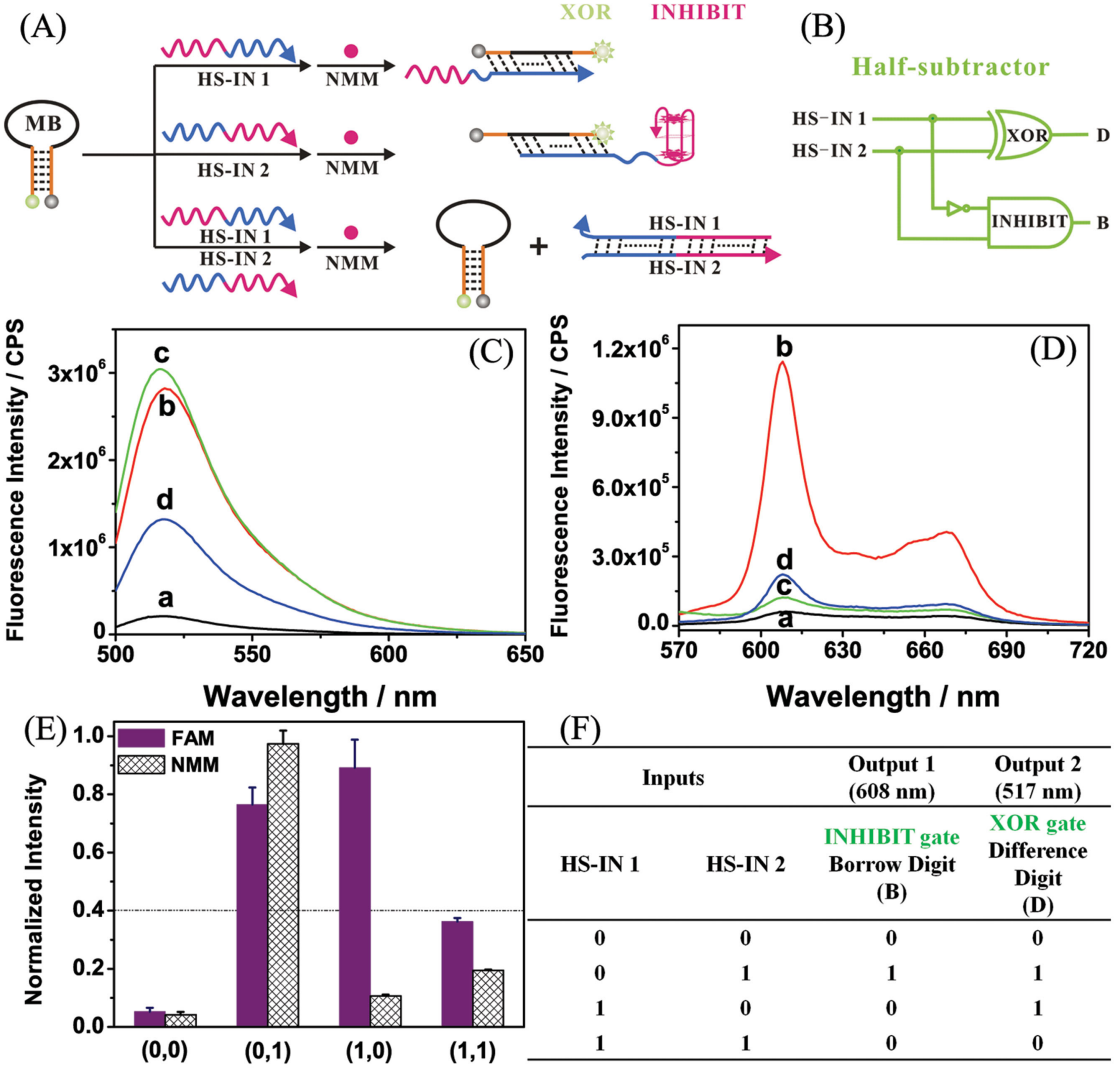
A) Schematic illustration of molecular‐scale implementation of a half subtractor based on DNA hybridization. B) Diagram of electronic half subtractor logic circuit. C,D) Fluorescence emission spectra of FAM (C) and NMM D) in the presence of a) MB; b) MB + HS‐IN 2; c) MB + HS‐IN 1; and d) MB + HS‐IN 1 + HS‐IN 2. E) Normalized fluorescence intensities of FAM and NMM outputs. F) Truth table for the half‐subtractor operation compiled from NMM‐based INH and FAM‐based XOR logic gates. FAM, 6‐carboxyfluorescein; NMM, *N*‐methyl mesoporphyrin IX; MB, molecular beacon; HS‐IN, half‐subtractor input.

Because all arithmetic functions, such as division, multiplication, exponentiation, can be implemented through integration of individual adders,^25^ a full adder was constructed. The principle of the present DNA‐based full‐adder operation is demonstrated in **Figure** **3**A. Ranked among the highest‐complexity in logic circuits, a full adder requires integration of two half adders and an additional OR logic gate to process three inputs (Figure 3B).^11^, ^26^ Until now, it has been a great challenge to build logic circuits to process three inputs at the molecular level.^26^, ^29^, ^31^ Here, the present full adder was based on the same MB platform to perform addition of three inputs (FA‐IN 1, FA‐IN 2 and FA‐IN 3 [CARRY IN, *C*_in_]). NMM and FAM fluorescence were also utilized as output signals, corresponding to CARRY OUT (*C*_out_) and SUM OUT (*S*_out_), respectively.

**Figure 3:**
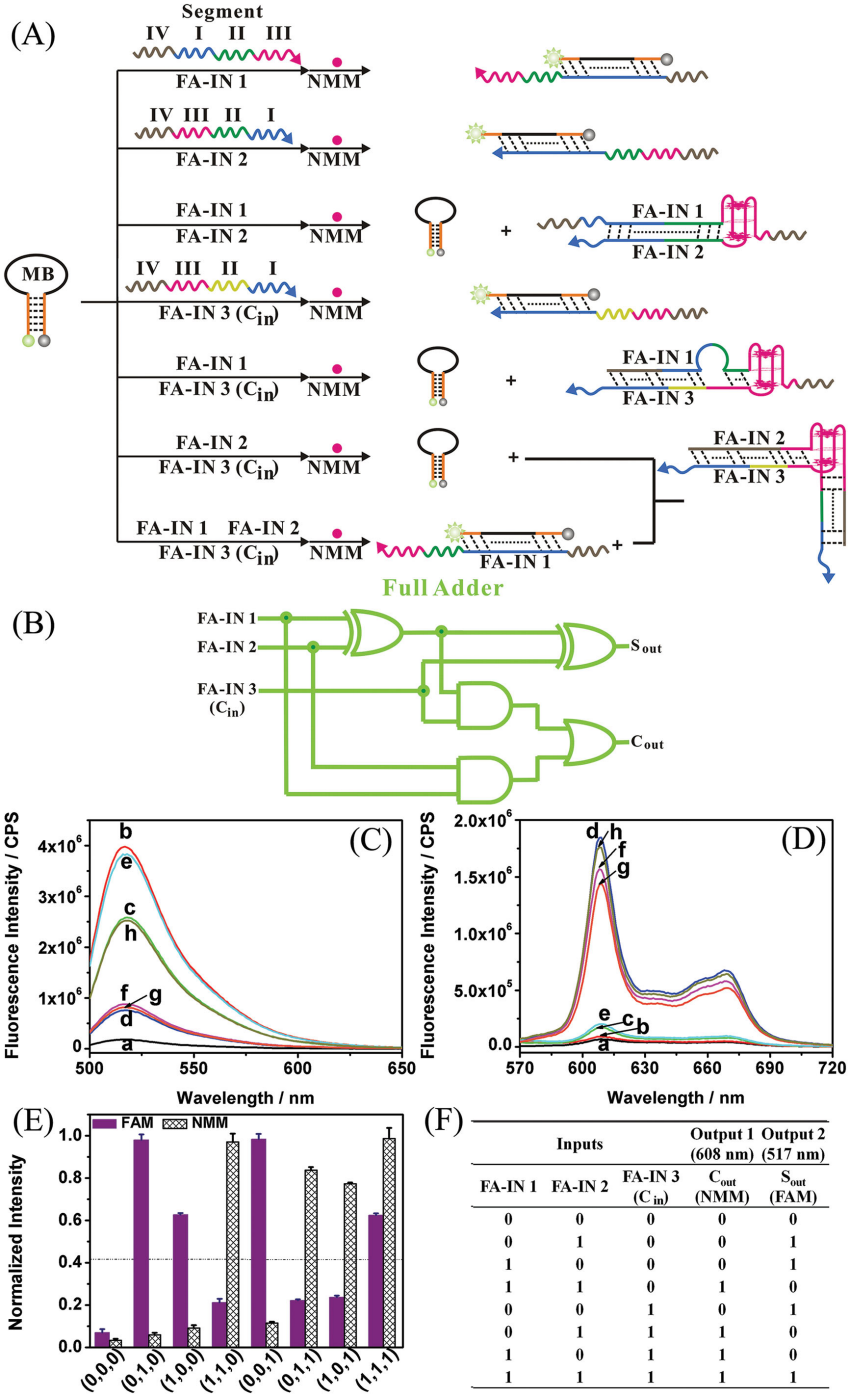
A) Schematic illustration of molecular‐scale implementation of a full adder based on DNA hybridization. B) Diagram of electronic full‐adder logic circuit. C,D) Fluorescence emission spectra of FAM (C) and NMM (D) in the presence of a) MB; b) MB + FA‐IN 2; c) MB + FA‐IN 1; d) MB + FA‐IN 1 + FA‐IN 2; e) MB + FA‐IN 3 (*C*_in_); f) MB + FA‐IN 2 + FA‐IN 3 (*C*_in_); g) MB + FA‐IN 1 + FA‐IN 3 (*C*_in_); h) MB + FA‐IN 1 + FA‐IN 2 + FA‐IN 3 (*C*_in_). E) Normalized fluorescence intensities of FAM and NMM outputs. F) Truth table for the full adder operation. FAM, 6‐carboxyfluorescein; NMM, *N*‐methyl mesoporphyrin IX; MB, molecular beacon; FA‐IN, full‐adder input; *C*_out_, CARRY OUT; *S*_out_, SUM OUT.

To construct a full adder, in general, each DNA strand input includes three functions; two functions are related to the generation of the two readout signals and the third function mediates the hybridization of DNA strands, favoring desired interactions while suppressing undesirable side reactions. To mediate the complicated interactions among the three inputs and the MB platform, a two‐base mismatch strategy was used to control hybridization energy and decrease secondary structure of the DNA input, directing the desired hybridization in a complex system. In brief, a two‐base mismatch was embedded in the sequence in each input that hybridizes to MB, which facilitates subsequent interaction in the presence of additional inputs through fully complementary hybridization. As shown in Figure 3A, each of the three inputs, FA‐IN 1, FA‐IN 2, and FA‐IN 3 (CARRY IN, *C*_in_), is able to open the MB hairpin. When any two of the three inputs are present at the same time, their mutual hybridization dominates the interactions in the system, inhibiting the hybridization of either input to MB, resulting in a weak FAM signal. Meanwhile, the hybridization of any two inputs can produce a G‐quadruplex from the respective G‐rich segments, resulting in a strongly fluorescent G‐4/NMM complex. In the presence of all three inputs, FA‐IN 2–3 hybridization is designed to dominate the reactions in the system and FA‐IN 1 is left free to hybridize with MB. The FAM and NMM fluorescence emission spectra in the presence of different input combinations are shown in Figure 3C,D, respectively. The discussion of changes in fluorescence change with different input combinations and PAGE analysis (Figure S3) is available in the Supporting Information. It is obvious that, after normalizing the fluorescence intensity (Figure 3E), a truth table (Figure 3F) fulfilling the requirement for a full adder operation is established according to the predefined threshold value. The truth table indicates eight arithmetic operations to be performed (FA‐IN 1 + FA‐IN 2 +*C*_in_): 0 + 0 + 0 = 0 (00), 0 + 1 + 0 = 1 (01), 1 + 0 + 0 = 1 (01), 1 + 1 + 0 = 2 (10), 0 + 0 + 1 = 1 (01), 0 + 1 + 1 = 2 (10), 1 + 0 + 1 = 2 (10), and 1 + 1 + 1 = 3 (11). Only the last operation is different from that for a half adder, where *C*_out_ = *S*_out_ = 1, corresponding to 1 + 1 + 1 = 3.^26^, ^29^

To further demonstrate the reconfigurability and scalability of the present system, a full subtractor was developed based on the same MB platform with stimulation by three newly designed inputs. Full subtractor is another logic circuit of the highest complexity^11^ and this is the first description of a full subtractor construction at the molecular level using DNA as building blocks.^29^, ^31^, ^58^ The principle underlying construction of the present full subtractor is illustrated in **Figure** **4**A. To precisely control the interaction among the DNA inputs and the MB platform, the two‐base mismatch strategy was also utilized in this design to decrease secondary structure, suppress generation of undesired byproducts, and facilitate the desired hybridization. In processing the three inputs (FS‐IN 1, FS‐IN 2, and FS‐IN 3 [BORROW IN, *B*_in_]), NMM and FAM fluorescence were again employed as the two output signals, serving as BORROW OUT (*B*_out_) and DIFFERENCE OUT (*D*_out_) digits, respectively. The complex logic circuit is shown in Figure 4B. A full subtractor integrates two half subtractors and an additional OR logic gate. Compared with a half subtractor, a full subtractor requires an additional input and executes subtraction of three inputs. A detailed description of interactions among the DNA strands, PAGE analysis (Figure S4, Supporting Information), and discussion of changes in fluorescence in the presence of different input combinations is available in the Supporting Information. To facilitate interpretation of the data, fluorescence emission spectra of FAM (Figure 4C) and NMM (Figure 4D) were normalized (Figure 4E), producing a corresponding truth table (Figure 4F) according to the predefined threshold. It should be noted that the normalized NMM fluorescence intensity is based on one equivalent G‐4/NMM complex in all logic operations. That is why the normalized fluorescence intensity of NMM is greater than 1 upon in the presence of inputs FS‐IN 2 and FS‐IN 3, where two equivalent G‐4/NMM complexes exist. There are eight arithmetic operations to be performed in a full subtractor circuit, as indicated in the truth table (Figure 4F). It should be noted that *D*_out_ takes into account an addition of 2 to the first input under conditions of a high borrow (*B*_out_ = 1), akin to a half subtractor. The detailed arithmetic operations (FS‐IN 1 – FS‐IN 2 – *B*_in_) are: (1) 0 – 0 – 0 = 0; (2) 0 – 1 – 0 = (‐1), which becomes 2 – 1 – 0 = 1 in the case of high borrow; (3) 1 – 0 – 0 = 1; (4) 1 – 1 – 0 = 0; (5) 0 – 0 – 1 = (‐1), it becomes 2 – 0 – 1 = 1 in the case of high borrow; (6) 0 – 1 – 1 = (‐2), changing into 2 – 1 – 1 = 0 in the case of high borrow; (7) 1 – 0 – 1 = 0; and finally, (8) 1 – 1 – 1 = (‐1), which becomes 3 – 1 – 1 = 1 in the case of high borrow.

**Figure 4:**
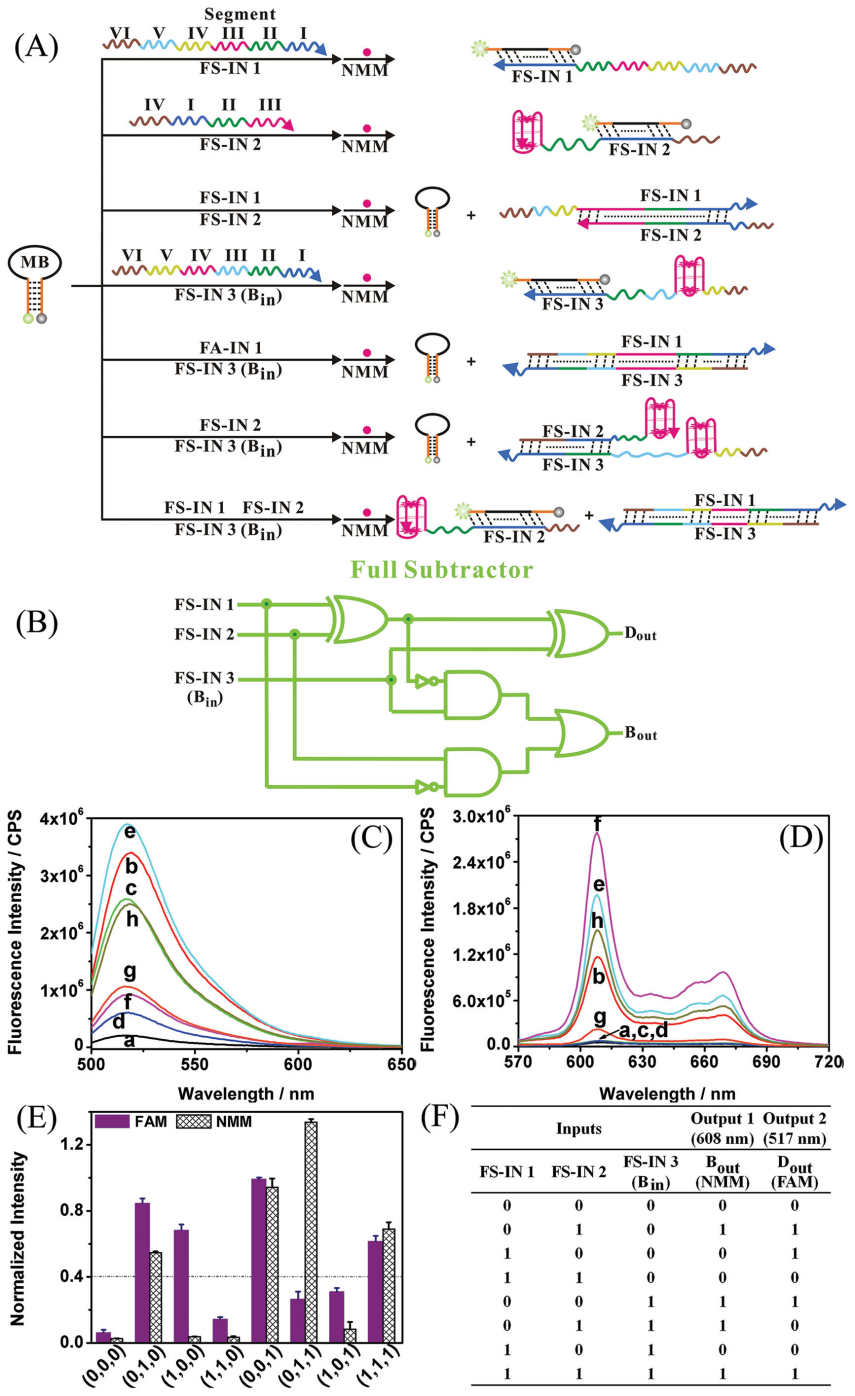
A) Schematic illustration of molecular‐scale implementation of a full subtractor based on DNA hybridization. B) Diagram of electronic full‐subtractor logic circuit. C,D) Fluorescence emission spectra of FAM (C) and NMM (D) in the presence of a) MB; b) MB + FS‐IN 2; c) MB + FS‐IN 1; d) MB + FS‐IN 1 + FS‐IN 2; e) MB + FS‐IN 3 (*B*_in_); f) MB + FS‐IN 2 + FS‐IN 3 (*B*_in_); g) MB + FS‐IN 1 + FS‐IN 3 (*B*_in_); h) MB + FS‐IN 1 + FS‐IN 2 + FS‐IN 3 (*B*_in_). E) Normalized fluorescence intensities of FAM and NMM outputs. F) Truth table for the full subtractor operation. FAM, 6‐carboxyfluorescein; NMM, *N*‐methyl mesoporphyrin IX; MB, molecular beacon; FS‐IN, full‐subtractor input; *B*_out_, BORROW OUT; *D*_out_, DIFFERENCE OUT.

For the sake of completeness, binary multiplication may be achieved because it follows the same rules as the truth of an AND gate. This is similar to binary division, which is the result of repeated subtraction (the same as decimal division).^26^ In all of the above parts, the positive logic convention has been utilized to construct the desired logic circuit, where “0” corresponds to the low output and “1” refers to the high output. In contrast, a negative logic convention holds for the opposite scenario by reversing the assignment, where a 0 corresponds to the high output and a “1” refers to the low output. The outputs and the inputs can take advantage of negative logic conventions. Therefore, negative logic has been widely used to increase output information channels for the molecular processor.^25^, ^29^ Here, a comparator can be further constructed by simply applying a negative logic gate to the above half subtractor (See Figure S5, Supporting Information).

In summary, a series of DNA‐based comprehensive logic circuits has been generated to perform arithmetic functions, including half adder, half subtractor, full adder, full subtractor, and a digital comparator. In comparison with previous studies,^25^, ^26^, ^27^, ^28^, ^29^, ^30^, ^31^, ^37^, ^38^, ^39^, ^40^, ^46^, ^59^ most of which are based on synthetic organic molecules, or are incomplete, or are built from different platforms, this is the first use of a simple and universal MB platform to construct these arithmetic logic circuits comprehensively, sharing the same threshold setpoint. In order to overcome obstacles related to the existence of high symmetry in sequence design, mismatched bases were embedded in the appropriate positions. In each arithmetic operation, dual outputs were generated in parallel stimulated by the same set of inputs. Furthermore, all signal outputs here are set in fluorescence mode, avoiding multi‐mode signal collection and, thus, simplifying operation. The developed enzyme‐free DNA systems have diverse advantages, including high flexibility, reconfigurability, massive parallelism, and scalability. The versatility and power of DNA computation demonstrated here indicate its great potential for solving complex combinatorial problems, competing with silicon‐based electronics in the long term. This system could also provide short‐term benefits in the biomedical field for disease diagnosis and therapy because of its biocompatibility.^36^, ^48^, ^60^, ^61^ Last but not least, DNA computation may aid in identifying the elusive mechanisms underlying some critical biological processes, a goal that far exceeds the concept of computation itself.^62^

## Supporting information

As a service to our authors and readers, this journal provides supporting information supplied by the authors. Such materials are peer reviewed and may be re‐organized for online delivery, but are not copy‐edited or typeset. Technical support issues arising from supporting information (other than missing files) should be addressed to the authors.

Supplementary
