# Supplementary material for: Implementation of Arithmetic Functions on a Simple and Universal Molecular Beacon Platform
Source: Adv Sci (Weinh). 2015 Apr 14;2(5):1500054. doi: 10.1002/advs.201500054 (PMC5115375; doi:10.1002/advs.201500054)
Supplement: Supplementary file 1 — Supplementary [file ADVS-2-0m-s001.pdf]

Copyright WILEY-VCH Verlag GmbH & Co. KGaA, 69469 Weinheim, Germany, 2015.

ADVANCED SCIENCE
Open Access

Supporting Information

for *Adv. Sci.*, DOI: 10.1002/advs.201500054

Implementation of Arithmetic Functions on a Simple and Universal Molecular Beacon Platform

Hailong Li, Shaojun Guo, Qinghui Liu, Lidong Qin, Shaojun Dong, Yaqing Liu,* and Erkang Wang*

Supporting Information

Implementation of Arithmetic Functions on a Simple and Universal Molecular Beacon Platform

Hailong Li, Shaojun Guo, Qinghui Liu, Lidong Qin, Shaojun Dong, Yaqing Liu, and Erkang Wang**

Materials. All chemicals used were of analytical grade and were used without further purification. Synthetic oligonucleotides were purchased from Shanghai Sangon Biotechnology Co. Ltd. (Shanghai, China) and TaKaRa Biotechnology (Dalian) Co. Ltd. (Dalian, China). The water used throughout all experiments was purified through a Millipore (Billerica, MA, USA) system. The stock solution of N-methyl mesoporphyrin IX (NMM, 1 mM) was prepared in dimethyl sulfoxide (DMSO) and stored in the dark at -20 ºC. The stock DNA solution was prepared with 25 mM Tris-HCl buffer (pH 7.0). DNA concentration was estimated by measuring the absorbance at 260 nm.

Instruments. A Varian (Palo Alto, CA, USA) Cary 500 Scan UV/Vis Spectrophotometer was used to quantify the oligonucleotides. Fluorescence intensities were recorded on a Fluoromax-4 spectrofluorometer (Horiba Jobin Yvon, Longjumeau, France).

DNA Sequence Design. Base mismatch was applied in the input sequence design to increase asymmetry and decrease secondary structure and undesired crosstalk. Upon application of base mismatch, it is possible to clearly control hybridization energy in the limited sequence design, where the concern of high symmetry exists, and drive the desired hybridization forward according to the difference in hybridization energy between duplexes. The hybridization energy can be described simply as Gibbs free energy in directing the reaction to formation of much more stable products. The change in free energy, as well as reaction trend, were estimated with the use of NUPACK (*Siam Review*, 2007, **49**, 65-88.), where coaxial stacking is not taken into consideration because of its complexity.

Spectrofluorimetry Studies. The desired concentration of NMM or DNA was obtained by diluting the corresponding stock solution with 10 mM Tris-HCl buffer containing 5 mM $MgCl_2$ and 15 mM KCl (pH 8.0), respectively. Each sample was denatured by heating at 95 ^{o}C for 5 min and then annealed by cooling to room temperature before addition of NMM. The concentration of DNA in each sample was 200 nM, and NMM concentration was fixed at 400 nM. The final volume of each sample for fluorescence measurement was 500 μL. To monitor

FAM fluroescence, excitation was carried out at 485 nm, and emission was monitored at 517 nm. Slit widths for excitation and emission in recording FAM spectra were set at 3 nm and 5 nm, respectively. For NMM fluorescence, excitation was carried out at 399 nm, and emission was monitored at 608 nm. Slit widths for both excitation and emission in NMM fluorescence recording were set at 5 nm. In normalizing the fluorescence intensity, the strongest fluorescence signal intensities of FAM (molecular beacon [MB]: 200 nM) and NMM (G-quadruplex [G-4]/NMM: 200 nM) in each arithmetic operation were set to "1". Error bars represent the standard deviation of three independent experiments.

Native Polyacrylamide Gel Electrophoresis. Polyacrylamide gels (12%) were prepared with 1×TBE buffer (89 mM Tris, 89 mM boric acid, 2 mM EDTA, pH 8.3). In each sample, the concentration of each DNA strand was 2 μM in 1×TBE buffer containing 12 mM Mg^{2+}. Each sample was denatured by heating at 95 ^{o}C for 5 min and then annealed by slow cooling to about 25 ^{o}C. Each sample (20 μL) was mixed with 2 μL of Gel-Dye Super Buffer Mix before loading onto the gel. The gel was run at a constant voltage of 100 V over a period of about 2.5 h. The gel was then immersed in 0.5 μg/mL ethidium bromide (EB) solution for about 1 h and then washed with water twice. The photographs were taken under UV light using a fluorescence imaging system (Vilber Lourmat, Marne-la-Vallée, France).

DNA sequence design

1. Half adder: design of DNA sequences

Sequences of DNA strands used in construction of the half adder (the mismatched base described in the text is indicated by lower case letter).

DNA	DNA sequence (From 5' to 3')
MB	FAM-GACTGT CGAC CTGCAG TGAT ACAGTC-Dabcyl
HA-IN 1	GGGT ATT AATG CTGG GACTGT ATaA CTGCAG GTA
HA-IN 2	TCA CTGCAG tTCG ACAGTC CCAG CATT AAT TGGGTGGGTGGG

2. Half subtractor: design of DNA sequences and discussion of associated results.

Sequences of DNA strands used in construction of the half subtractor (the mismatched base described in the text is indicated by lower case letter).

DNA	DNA sequence (From 5' to 3')
MB	FAM-GACTGT CGAC CTGCAG TGAT ACAGTC-Dabcyl
HS-IN 1	CCCACCCACCCACCCA ATT AATG CTGG GACTGT ATaA CTGCAG GTA
HS-IN 2	TCA CTGCAG tTCG ACAGTC CCAG CATT AAT TGGGTGGGTGGGTGGG

Through careful design of the input sequence, either HS-IN 1 or HS-IN 2 is able to open the hairpin structure upon hybridization with MB, resulting in separation of FAM from the Dabcyl quencher and thus recovery of FAM fluorescence. Similar to the half adder design shown above, a single-base mismatch is also embedded in the duplex section of both the MB−HS-IN 1 and MB−HS-IN 2 complexes. The mismatched base is indicated with lower case letter in each input sequence. When both inputs are present, the HS-IN 1-2 complex forms because it is more stable than MB−HS-IN 1 or MB−HS-IN 2, and a weak FAM fluorescence signal is observed. With respect to the NMM output signal, only HS-IN 2 is designed to form a G-quadruplex alone from its free G-rich terminal, resulting in the highly fluorescent G-4/NMM complex. These interaction were validated by PAGE analysis (see Figure S2), further confirming the successful design of the interacting DNA strands for construction of the half subtractor.

The fluorescence spectra of FAM in Figure 2C and NMM in Figure 2D with different input combinations confirm the feasibility of constructing a half subtractor. Either input leads to high FAM fluorescence (curves b and c in Figure 2C). FAM fluorescence is much weaker in the absence or presence of both inputs (curves a and d, respectively, in Figure 2C). Consequently, the FAM-based XOR logic gate was successfully fabricated. Meanwhile, a strong NMM fluorescent signal is observed in the presence of MB and HS-IN 2 alone due to the formation of the G-4/NMM complex (curve b in Figure 2D). HS-IN 1 cannot form a G-quadruplex by itself and can inhibit G-quadruplex formation by hybridizing with HS-IN 2, leading to weak NMM fluorescence (curves c and d, respectively, in Figure 2D). Thus, a NMM-related INHIBIT logic gate was achieved. After normalizing the fluorescence intensities of FAM and NMM (shown in Figure 2E), a truth table can be obtained (Figure 2F).

3. Full adder: design and interactions of DNA sequences

Sequences of DNA inputs used in the operation of the full adder (two-base mismatches described in the text are indicated by lower case letters).

DNA	DNA sequence (From 5' to 3')
MB	FAM-GACTGT CGAC CTGCAG TGAT ACAGTC-Dabcyl
FA-IN 1	AC CTG ac G TGAT ACAG (Segment IV) GTATCA CTGCAG TgcG ACAGTC (Segment I)<br>ACCA AGGA TATAAC (Segment II) TTTGGGTGGG (Segment III)
FA-IN 2	CTG ac GTGAT ACAG GT AT CACT GC (Segment IV) TATAAC TTT GGGT GGGT TT (Segment III)<br>GTTATA TCCT TGGT (Segment II) GACTGT CgcA CTGCAG GTCGAC (Segment I)
FA-IN 3 (C_{in})	GCAG TgcG ACAGTC ACCA AGGA TATAAC (Segment IV) aaT GGG TGGG Taa GTTATA (Segment III)<br>GCAGTGAT AC (Segment II) CTGT ATCA Cgt CAG GTCG ACAG (Segment I)

Each of the three inputs, FA-IN 1, FA-IN 2 and FA-IN 3 (CARRY IN, C_{in}), is able to open the molecular beacon through hybridization between MB and its Segment I (where two-base mismatch is embedded), resulting in a strong FAM fluorescence signal (curves b, c, e in Figure 3C), with lack of formation of fluorescent G-4/NMM complex (curves b, c, e in Figure 3D). When any two of the three inputs are present simultaneously, their mutual hybridization dominates the interactions of the system, and inhibits the hybridization of any input to MB, leading to a weak FAM signal. Meanwhile, the hybridization of any two inputs can produce a G-quadruplex from the respective G-rich segments, resulting in strongly fluorescent G-4/NMM complex. The presence of FA-IN 1 and FA-IN 2 results in partially hybridization through part of Segment I and Segment II in FA-IN 1 complementary to part of Segment I and Segment II in FA-IN 2. The formation of the FA-IN 1-2 complex inhibits the interaction of either input with MB and facilitates G-quadruplex generation from the G-rich Segment III of both FA-IN 1 and FA-IN 2, resulting in weak FAM fluorescence (curve d in Figure 3C) and strong NMM fluorescence due to formation of G-4/NMM complex (curve d in Figure 3D). The interaction between FA-IN 1 and FA-IN 3 occurs in three steps to guarantee FA-IN 1-3 complex formation: (1) Segment IV in FA-IN 1 binds to the complementary region of Segment I in FA-IN 3; (2) Segment II in FA-IN 3 binds to the complementary region of Segment I in FA-IN 1; (3) Partially complementary duplex and G-quadruplex formation between Segments II and III in FA-IN 1 and Segment III in FA-IN 3. Steps (1) and (2) are consecutive, suppressing hybridization of either FA-IN 1 or FA-IN 3 to MB and thus generating weak FAM fluorescence (curve g in Figure 3C). Duplex formation in (3) favors formation of the G-quadruplex, resulting in strong NMM fluorescence (curve g in Figure 3D). In the last case of two inputs, the interaction between FA-IN 2 and FA-IN 3 also consists of three steps: two consecutive hybridizations to form two duplexes and embedding of a G-

quadruplex between them. One duplex forms in a region between Segment IV and part of Segment III in FA-IN 2 that is complementary to a region spanning part of Segment I, Segment II and part of Segment III in FA-IN 3. The other duplex forms at site spanning part of Segment I, Segment II and part of Segment III in FA-IN 2 that is complementary to sequence spanning part of Segment III and Segment IV in FA-IN 3. Formation of these two duplexes inhibits the interaction of the respective input with MB, generating weak FAM fluorescence (curve f in Figure 3C). The G-rich Segment III in both FA-IN 2 and FA-IN 3 lead to G-quadruplex formation and strong NMM fluorescence (curve f in Figure 3D). In the presence of all three inputs, FA-IN 2-3 hybridization is designed to dominate the reactions of the system and FA-IN 1 is left alone to hybridize with MB and separate FAM from Dabcyl quencher, finally producing strong NMM (curve h in Figure 3D) and FAM fluorescence (curve h in Figure 3C), respectively. This interaction was confirmed by PAGE analysis (see Figure S3).

4. Design of full subtractor and discussion of associated results.

Sequences of DNA inputs used in the operation of a full subtractor (two-base mismatches described in the text are indicated by lower case letters).

DNA	DNA sequence (From 5' to 3')
MB	FAM-GACTGT CGAC CTGCAG TGAT ACAGTC-Dabcyl
FS-IN 1	CGAC CTttAG TGAT ACAG (Segment VI) GT ATCA CTGCAG TGC (Segment V) AAAAAAAA AAAAA (Segment IV)<br>A CCCA CCCA CCCA CCCA (Segment III) AAAAAAAAAAAAA (Segment II) GACTGTCgcA CTGCAG GTCGAC (Segment I)
FS-IN 2	GT CGAC CTTTAG TGAT ACAG (Segment IV) GT ATCA CTGCAG TgcG ACAGTC (Segment I)<br>TTTTT TTTT TTTT (Segment II) TGGG TGGG TGGG TGGG (Segment III)
FS-IN 3 (B_{in})	GCAG TGCG ACAGTC (Segment VI) TTTT TTTT TTTTT (Segment V) TGGG TGGG TGGG TGGGT (Segment IV)<br>TTTTT TTTT TTTT (Segment III) GCA CTGCAG TGAT AC (Segment II) CTGT ATCA CTaaAG GTCG ACAG (Segment I)

The detailed interaction among the DNA strands can be clearly seen in Figure 4A and related supporting information. Each input DNA strand is able to hybridize with the MB platform (a two-base mismatch is embedded in each hybridizing sequence), separating FAM from the Dabcyl quencher and thus generating a highly fluorescent FAM signal (curves b, c, e in Figure 4C). Meanwhile, either FS-IN 2 or FS-IN 3 can form a G-quadruplex due to their respective G-rich segment (Segment III in FS-IN 2 and Segment IV in FS-IN 3), generating a

G-4/NMM complex and strong NMM fluorescent signal (curves b and e in Figure 4D). In contrast, only weak NMM fluorescence was observed in the presence of FS-IN 1 alone because this input alone is incapable of forming a G-quadruplex (curve c in Figure 4D). In the presence of any two inputs, mutual hybridization is designed to dominate the reactions in the system, and MB is not affected. The simultaneous presence of FS-IN 1 and FS-IN 2 leads to a partially hybridized complex through Segments I, II, III in both inputs. Part of Segment I in FS-IN 1 is fully complementary to part of Segment I in FS-IN 2, which inhibits hybridization of the MB platform to either input and is accompanied by weak FAM fluorescence (curve d in Figure 4C). This inhibition is further increased through hybridization between other sequences in both inputs and the consecutive nature of these hybridizations. Segment III in FS-IN 1 inhibits G-quadruplex formation by Segment III in FS-IN 2 due to the full complementarity of the two sequences, this results in lack of formation of a fluorescent G-4/NMM complex (curve d in Figure 4D). Hybridization between Segment II in FS-IN 1 and FS-IN 2 enhances the mutual interaction, further suppressing G-4/NMM complex formation as well as the reaction of each input with the MB platform. In the presence of FS-IN 2 and FS-IN 3 together, the interaction between them that results in the formation of the FS-IN 2-3 complex consists of two steps: (1) Segment IV in FS-IN 2 binds to complementary sequence in part of Segment I of FS-IN 3, and (2) part of Segment I in FS-IN 2 binds to complementary Segment II in FS-IN 3. The consecutive nature of the hybridizations that forms the FS-IN 2-3 complex can block the formation of MB−FS-IN 2 and MB−FS-IN 3, where G-quadruplex formation by the G-rich Segment III of FS-IN 2 or Segment IV of FS-IN 3 is not affected. Thus, a weak FAM fluorescence signal is observed (curve f in Figure 4C), and a strong NMM fluorescence signal can be detected due to the formation of the G-4/NMM complex (curve f in Figure 4D). In the last case of two inputs, FS-IN 1 and FS-IN 3, the sequence spanning part of Segment I through Segment VI in FS-IN 1 binds to the fully complementary sequence in FS-IN 3 that spans Segment VI through partial Segment I. Sequence spanning Segment VI and part of Segment I in FS-IN 1 binds to complementary sequence spanning part of Segment I and Segment VI in FS-IN 3, respectively, which hinders the interaction between either input and MB facilitated by sequential formation of other duplexes within the FS-IN 1-3 complex, producing weak FAM fluorescence (curve g in Figure 4C). Formation of a highly fluorescent G-4/NMM complex by the G-rich Segment IV of FS-IN 3 in combination with NMM is also blocked by hybridization of Segment IV in FS-IN 3 to Segment III in FS-IN 1 (curve g in Figure 4D). Other duplexes are designed to increase the tendency toward generation of the two desired fluorescent products and guarantee that the reaction between FS-IN 1 and FS-IN

3 dominates the system in the presence of all three inputs, where the FS-IN 1-3 and MB−FS-IN 2 complexes are the main products. Accordingly, strong FAM and NMM fluorescence signals are obtained simultaneously in the presence of all three inputs (curve h in Figure 4C and 4D, respectively). The interaction among all DNA strands associated with the present full subtractor was further confirmed by PAGE analysis (Figure S4).

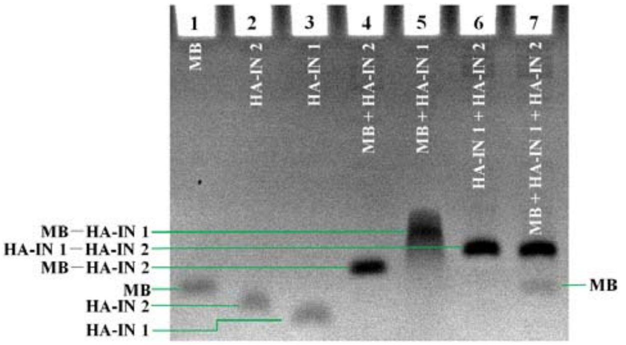

Figure S1. Native polyacrylamide gel analysis of the interactions among MB, HA-IN 1, and HA-IN 2 DNA strands. The sample in each lane and the identities of the main bands are indicated.

Discussion of Figure S1: The new bands in lanes 4 and 5 indicate the formation of MB−HA-IN 2 and MB−HA-IN 1 complexes, respectively. The presence of all three DNA strands generates two bands (lane 7), which are ascribed to HA-IN 1-2 complex (as in lane 6) and MB.

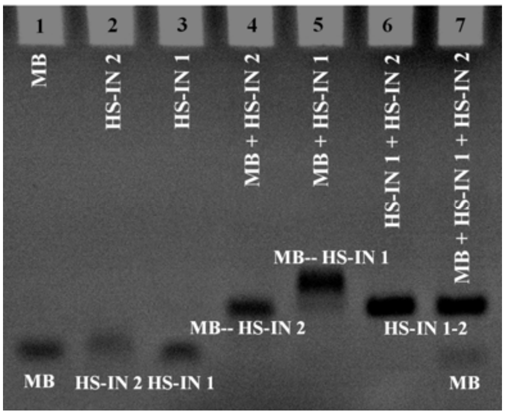

Figure S2. Native polyacrylamide gel analysis of the interactions among MB, HS-IN 1, and HS-IN 2 DNA strands. The sample in each lane and the identities of the main bands are indicated.

Discussion of Figure S2: The new bands in lanes 4 and 5 indicate the formation of complexes MB−HS-IN 2 and MB−HS-IN 1, respectively. The presence of all three DNA strands generates two bands (lane 7), which are ascribed to HS-IN 1-2 complex (as in lane 6) and MB.

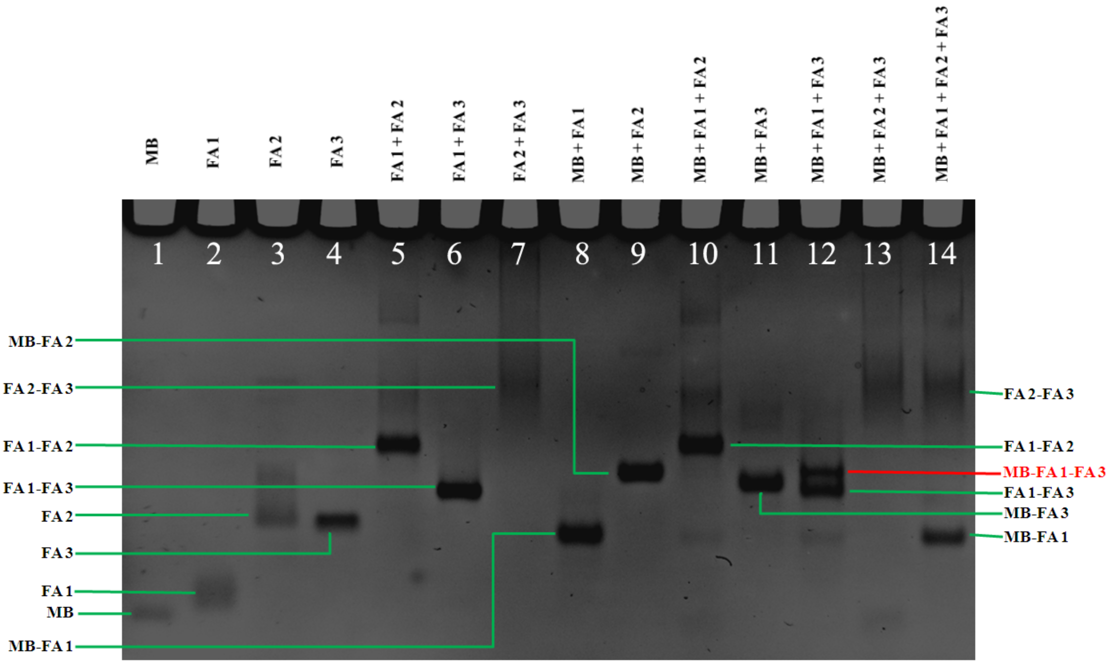

Figure S3. Native polyacrylamide gel analysis of the interactions among MB, FA-IN 1, FA-IN 2, and FA-IN 3 (C_{in}) DNA strands. The sample in each lane and the identities of the main belts are indicated above and at the sides of the gel image, respectively. FA 1, FA-IN 1; FA 2, FA-IN 2; FA 3, FA-IN 3; C_{in}, CARRY IN.

Discussion of Figure S3: The bands in lanes 1, 2, 3, and 4 correspond to MB, FA-IN 1, FA-IN 2, and FA-IN 3 (C_{in}), respectively. The bands in lanes 5, 6, and 7 indicate the formation of FA-IN 1-2, FA-IN 1-3, and FA-IN 2-3 complexes, respectively. The band smearing in lane 7 may be caused by the G-quadruplex, embedded in the middle of FA-IN 2-3 complex (J. Am. Chem. Soc. **2012**, *134*, 3508−3516). Bands in lanes 8, 9, and 11 indicate formation of stable complexes MB−FA-IN 1, MB−FA-IN 2, and MB−FA-IN 3, respectively. However, in the presence of MB and two inputs, the interaction between the inputs dominates the reactions of the system and only the complex formed by the two inputs is observed, as indicated in lanes 10, 12, and 13. In lane 12, the band above FA-IN 1-3 band may be ascribed to the byproduct MB−FA-IN 1-3. In the presence of MB and three inputs (lane 14), two bands are present, corresponding to FA-IN 2-3 and MB−FA-IN 1 complexes. In some cases, the unreacted MB band cannot be discerned, which may be caused by the low density of the MB band itself, as well as formation of some unexpected byproducts (lanes 10, 12, and 13).

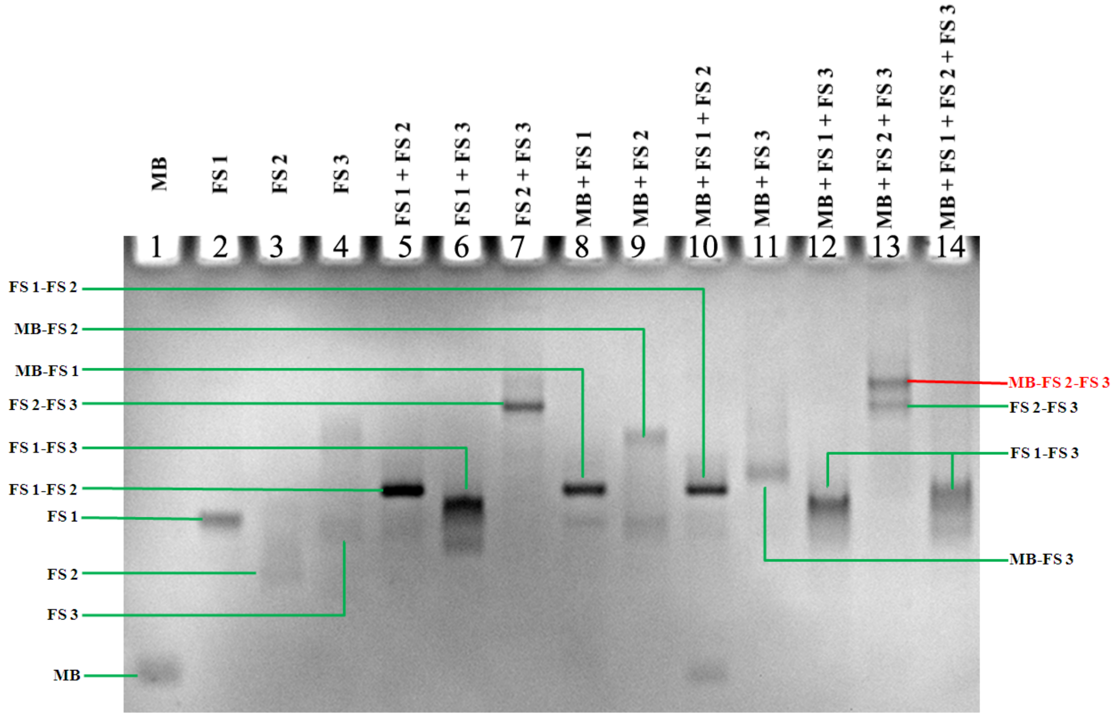

Figure S4. Native polyacrylamide gel analysis of the interactions among MB, FS-IN 1, FS-IN 2, and FS-IN 3 (B_{in}) DNA strands. The sample in each lane and the identities of the main bands are indicated above and at the sides of the gel image, respectively. FS 1, FS-IN 1; FS 2, FS-IN 2; FS 3, FS-IN 3; B_{in}, BORROW IN.

Discussion of Figure S4: Bands in lanes 1, 2, 3, and 4 correspond to MB, FS-IN 1, FS-IN 2, and FS-IN 3 (B_{in}), respectively. The bands in lanes 5, 6, and 7 indicate the formation of complexes FS-IN 1-2, FS-IN 1-3, and FS-IN 2-3, respectively. The band smearing in lanes 3 and 4 may be caused by the generated G-quadruplex (J. Am. Chem. Soc. **2012**, *134*, 3508−3516). Bands repersenting the sable complexes MB−FS-IN 1, MB−FS-IN 2, and MB−FS-IN 3 can be seen in lanes 8, 9, and 11, respectively. Some smearing, also due to G-quadruplex formation, is seen in lanes 9 and 11. However, in the presence of MB and two inputs, the interaction between the inputs dominates the reactions in the system and only the complex formed by the two inputs is observed, as indicated in lanes 10, 12, and 13. In lane 13, the band above the FS-IN 2-3 band may be ascribed to the byproduct MB−FS-IN 2-3, which is one reason for the low density of the FS-IN 2-3 band. The smearing of the FS-IN 2-3 band due to inclusion of the G-quadruplex within the duplex also contributes to its low density. In the presence of three inputs and MB (lane 14), a band corresponding to the FS-IN 1-3 complex can be observed. The expected MB−FS-IN 2 band is undetectable due to smearing, which can be seen in lane 9. In some cases, the band representing unreacted MB is not clear,

which may be due to the low density of the band, as well as to formation of some unexpected byproducts (lanes 12 and 13).

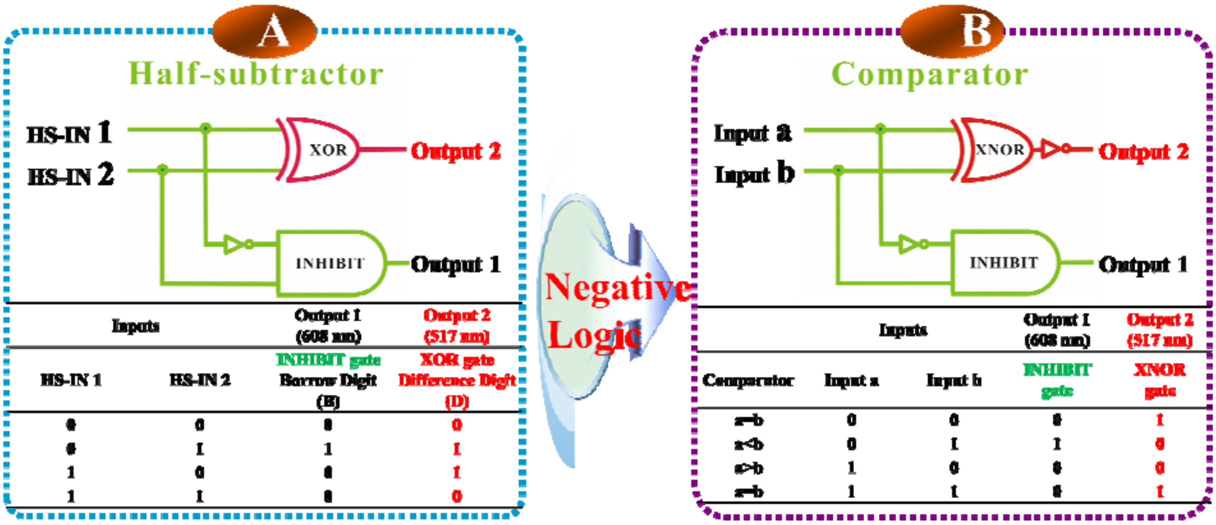

Figure S5. Application of negative logic in construction of a comparator. (**A**) Logic circuit and truth table of the pre-constructed half-subtractor. (**B**) Logic circuit and truth table of a comparator generated by application of negative logic to the half-subtractor. HS-IN 1 and HS-IN 2 correspond to Input a and Input b, respectively. HS-IN, half-subtractor input.

As discussed above, we have succeeded in building a half-subtractor based on the simple MB platform, as shown in Figure S5A. If a negative logic convention is applied to Output 2, an XNOR function is then achieved. In combination with the INHIBIT function (Output 1), a comparator is successfully implemented, which can be clearly learned from the truth table (Figure S5B) (*J. Phys. Chem. C* **2008**, *112*, 7047-7053). In general, there are two basic types of digital comparators: identity comparators and magnitude comparators. Identity comparators are used to determine whether two inputs are equal, corresponding to the XNOR logic gate in Figure S5B. Magnitude comparators are able to determine the relative magnitude of two inputs, determining which input is larger or smaller. Therefore, a molecular digital comparator can be constructed by applying a negative logic convention to FAM outputs and a positive logic convention to the NMM outputs. Herein, the comparison between *a* and *b* can be seen from the truth table in Figure S5B. The state of “1” in Output 2 of the comparator represents the equality of *a* and *b* (*a*=*b*). If Output 1 is “1”, then $b > a$. Under the condition that both Output 1 and Output 2 are “0”, it can be determined that $b < a$. In conclusion, a digital comparator has been successfully constructed through simple application of a negative logic convention to the above constructed half subtractor.
